# Supplementary material for: Endothelial IGF‐1 receptor mediates crosstalk with the gut wall to regulate microbiota in obesity
Source: EMBO Rep. 2021 May 2;22(5):e50767. doi: 10.15252/embr.202050767 (PMC8097321; doi:10.15252/embr.202050767)
Supplement: Supplementary file 1 — Expanded View Figures PDF [file EMBR-22-e50767-s005.pdf]

Expanded View Figures

Figure EV1. Endothelial IGF-1R overexpression has no effect on glucose tolerance in chow-fed mice.

A, B In aorta from human IGF-1R endothelial overexpressing mice (hIGFREO) and wild-type littermates (WT) after 8 weeks HFD, immunoblotting shows that hIGFREO have similar protein expression of phospho-eNOS at Serine 1177 and phospho-AKT at Serine 473 (n = 8–10 mice per group).

C hIGFREO had comparable glucose fasting blood glucose levels compared with WT on chow diet (n = 5–10 mice per group).

D, E hIGFREO had comparable glucose intolerance, compared to WT on chow diet (as measured by glucose tolerance test and area under the curve (AUC)) (5–10 mice per group).

F, G In muscle from WT and hIGFREO after 8 weeks of HFD, immunoblotting shows that hIGFREO have increased protein expression of total and phospho-AKT at Serine 473 (n = 3 per genotype).

Data information: Data shown as mean ± SEM and individual mice are shown as data points, $P < 0.05$ taken as being statistically significant using Student's t-test. ns denotes not significant.
Source data are available online for this figure.

© 2021 The Authors

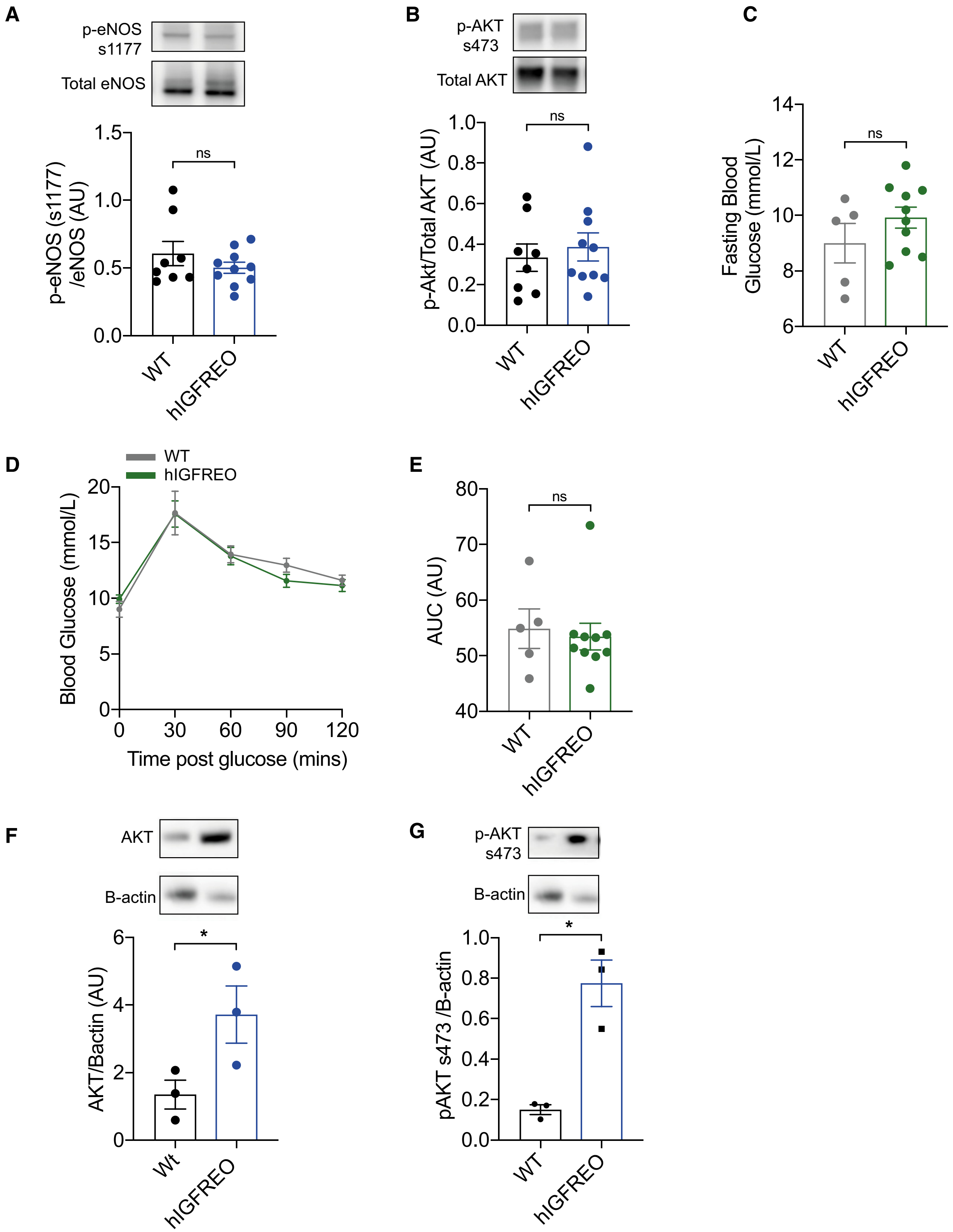

Figure EV1.

 © 2021 The Authors

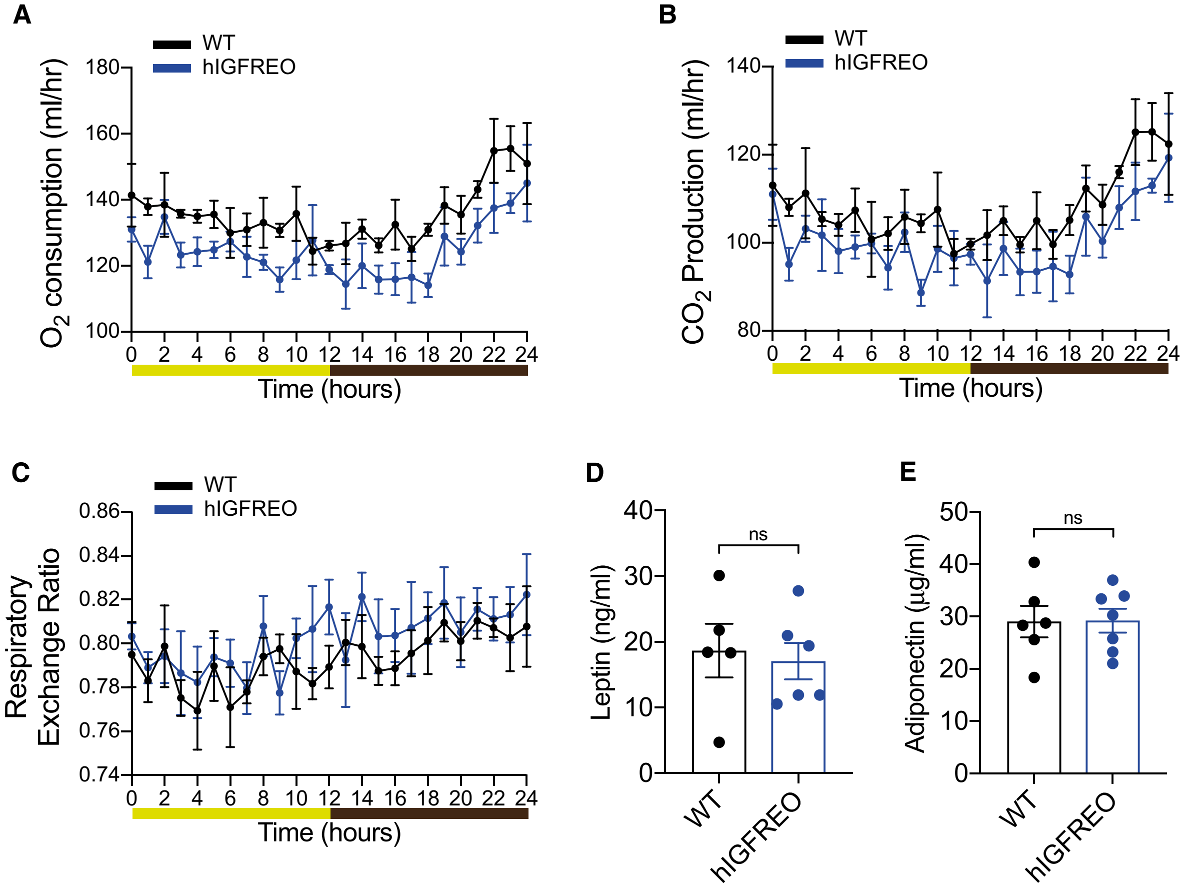

Figure EV2. Protection from high-fat diet (HFD)-induced weight gain is not due to altered energy expenditure in mice overexpressing human IGF-1R in the endothelium (hIGFREO).

A–C hIGFREO exhibit no difference in oxygen consumption, CO_2 production or respiratory exchange ratio using indirect calorimeter assessment after HFD when compared to WT after 8 weeks of HFD (n = 4 per genotype).

D, E hIGFREO had comparable concentrations of fasting plasma leptin and adiponectin to WT after 8 weeks of HFD (n = 5–7 mice per group).

Data information: Data shown as mean ± SEM and individual mice are shown as data points, $P < 0.05$ taken as being statistically significant using Student's t-test and denoted as * and ns denotes not significant. For indirect calorimetry, ANOVA testing was performed using mass as a co-variant (ANCOVA testing) using calrapp.org.

Figure EV3. Adipose remodelling was no different in human IGF-1 receptor endothelial overexpressing mice (hIGFREO) in the setting of high-fat diet (HFD)-induced obesity.

A–C (A), Histological examination of brown and white adipose tissue in hIGFREO compared with WT after 8 weeks of HFD. Scale bar = 300 µm. (B), hIGFREO mice have increased abundance of smaller adipocytes than WT. (C), There is no difference in average size of adipocytes in brown adipose tissue of hIGFREO compared with WT using haematoxylin and eosin stain (n = 6 per genotype).

D Representative confocal microscopy images of whole mount white and brown adipose tissue. Adipocytes stained with LipidTOX (green) and endothelial cells stained with Isolectin B4 (IB4-647) (red). Scale bar = 50 µm (n = 4–5 mice per group).

E There is no difference in vascular density in white or brown adipose tissue from hIGFREO compared with WT after 8 weeks of HFD (7 per genotype).

F Macrophage infiltration, shown by crown-like structure (CLS) analysis, was similar in hIGFREO and WT after 8 weeks of HFD (n = 7–8 mice per group).

G–I There is also no difference in resident adipose $CD45^+$ cells, $CD11b^+$ cells or F4/80 in hIGFREO compared with WT after 8 weeks of HFD (n = 7–8 mice per group).

Data information: Data shown as mean ± SEM and individual mice are shown as data points, $P < 0.05$ taken as statistically significant using Student's t-test and denoted as * and ** for $P < 0.01$. ns denotes not significant.

© 2021 The Authors

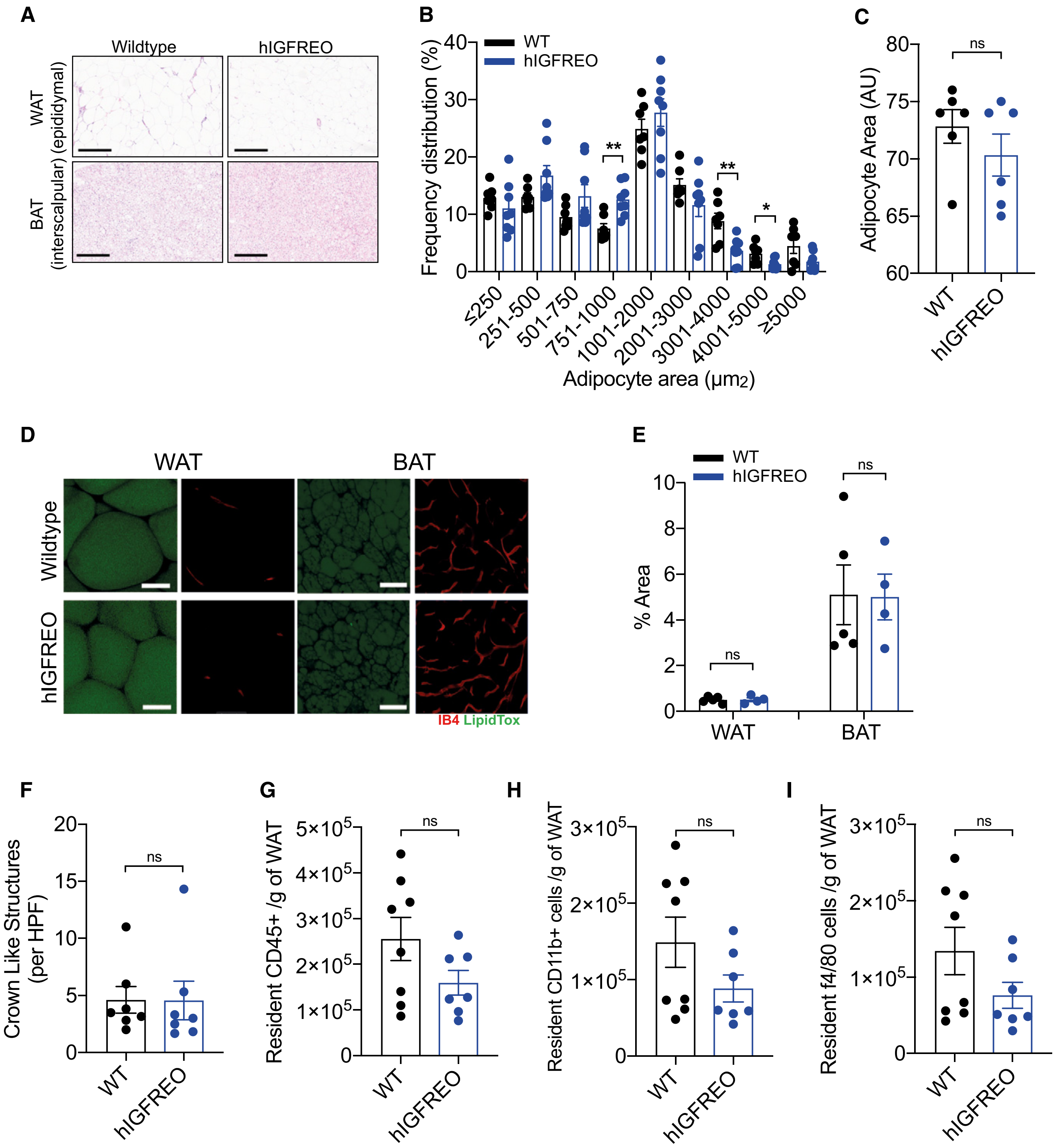

Figure EV3.

 © 2021 The Authors

Figure EV4. There was no difference in hepatic steatosis in human IGF-1 receptor endothelial overexpressing mice (hIGFREO) in the setting of high-fat diet-induced obesity.

A Representative images of haematoxylin- and eosin-stained liver from hIGFREO compared with wild-type littermates (WT). Scale bar = 200 µm.
B No difference in extent of hepatic fibrosis in hIGFREO compared with WT after 8 weeks of HFD (n = 7–9 mice per group).
C This was confirmed using MR images showing that there was no difference in hepatic fat content when comparing hIGFREO to WT after 8 weeks of HFD (n = 4 per genotype).
D–F Hepatic levels of free fatty acids, triglycerides and cholesterol are similar when comparing hIGFREO to WT after 8 weeks of HFD (n = 7–11 mice per group).
G, H Fasted plasma concentration of free fatty acids and triglycerides is also similar in hIGFREO and WT (n = 4–8 mice per group).
I, J (I) Pancreatic lipase levels and lipase activity (J) were similar in hIGFREO and WT after 8 weeks of HFD (n = 4–6 mice per group).
K Hepatic expression of CYP7A and ABCB11 is also similar in hIGFREO and WT after 8 weeks of HFD (n = 5–6 mice per group).

Data information: Data shown as mean ± SEM and individual mice are shown as data points, $P < 0.05$ taken as being statistically significant using Student's t-test. ns denotes not significant.

© 2021 The Authors

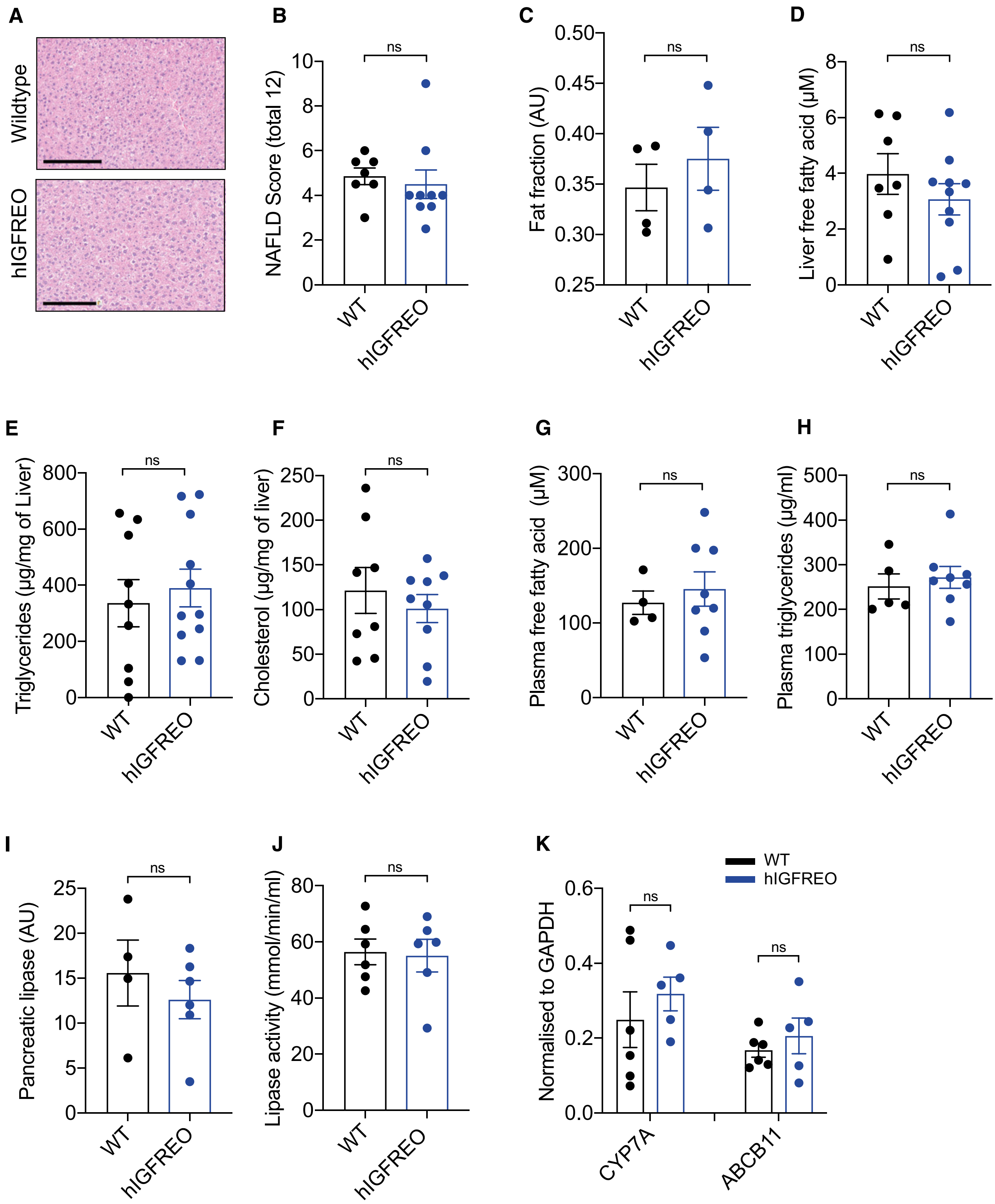

Figure EV4.

 © 2021 The Authors

Figure EV5. Endothelial cells from human IGF-1 receptor endothelial overexpressing mice (hIGFREO) can communicate with the gut wall.

A–C (A), Representative images of haematoxylin- and eosin-stained villi from hIGFREO compared with WT after 8 weeks of HFD. No difference in villi lipid content (B) or villi length (C) in hIGFREO compared with WT (n = 5–12 mice per group).

D, E Faith's phylogenetic diversity (PD) was used to measure the faecal microbial diversity and demonstrates no difference between hIGFREO mice and WT on chow diet (n = 5-8 mice per group).

F, G Chao-1 analysis was used to measure the faecal microbial diversity and abundance and again demonstrates no difference between hIGFREO and WT mice on chow diet (n = 5–8 mice per group).

H–J Targeted gene expression of the small intestine from hIGFREO normalised to WT after 8 weeks of high-fat diet demonstrating a significant increment in Muc2 and decrement in Cd36, Sar1b, Apob and Defb1 (n = 6–8 mice per group).

K Gene expression of Caco-2 cells treated with conditioned media from hIGFREO endothelial cells showed a significant increase in Reg3g expression (n = 3–6 mice per group).

Data information: Data shown as mean ± SEM and individual mice are shown as data points, $P < 0.05$ taken as being statistically significant using Student's t-test and denoted as *. Ns denotes not significant. Diversity analyses were run on the resulting OTU/feature.biom tables to provide both phylogenetic and non-phylogenetic metrics of alpha and beta diversity. Additional data analysis (PLS-DA) and statistics were performed with R.

© 2021 The Authors

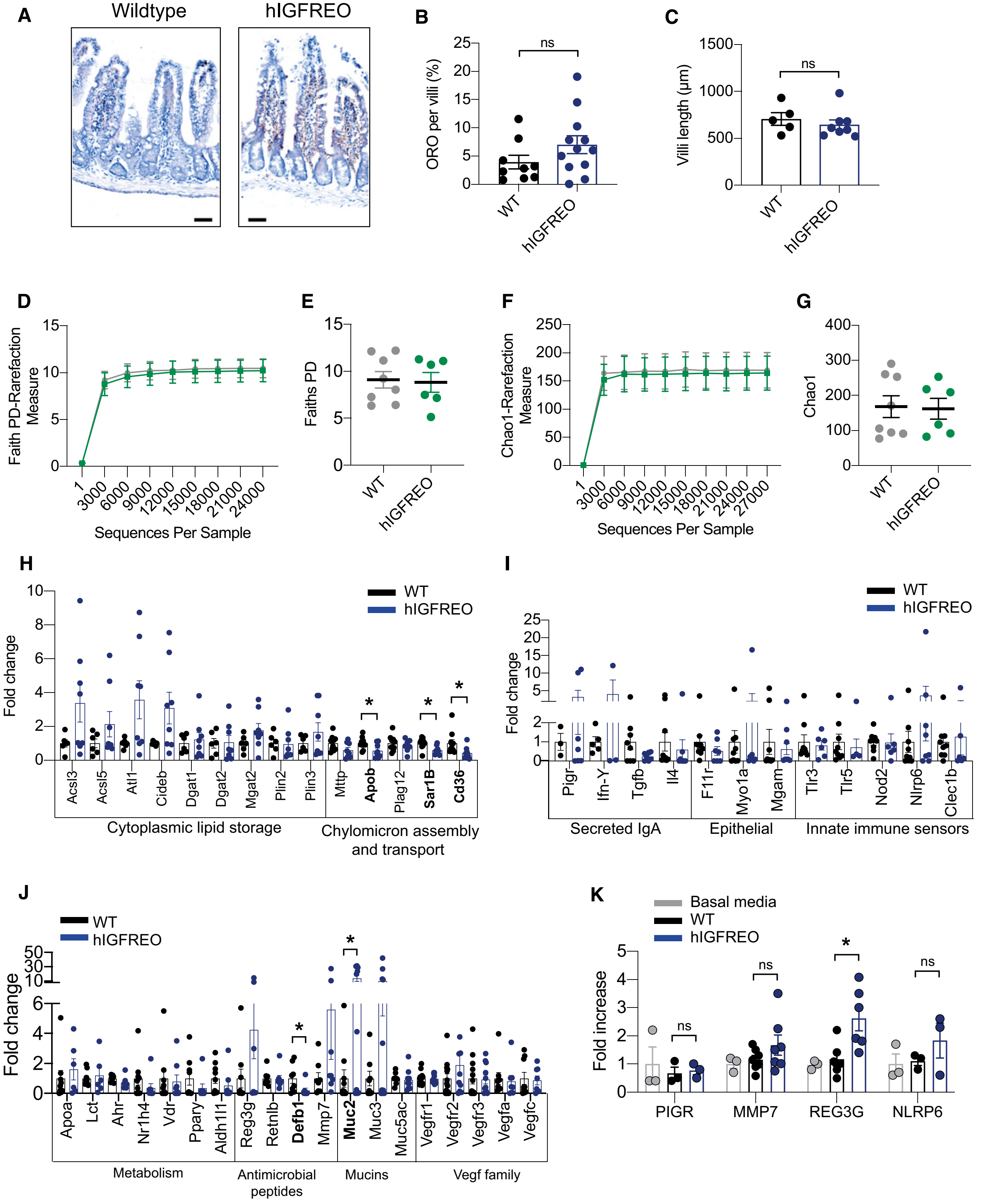

Figure EV5.

© 2021 The Authors
