# Supplementary material for: Endothelial IGF‐1 receptor mediates crosstalk with the gut wall to regulate microbiota in obesity
Source: EMBO Rep. 2021 May 2;22(5):e50767. doi: 10.15252/embr.202050767 (PMC8097321; doi:10.15252/embr.202050767)
Supplement: Supplementary file 2 — Table EV1 [file EMBR-22-e50767-s002.docx]

|  | WT  + Abs | hIGFREO  + Abs |
| --- | --- | --- |
| (f)Peptococcaceae;(g)Peptococcus | 0 | 0 |
| (f)Enterobacteriaceae;(g)Escherichia Shigella | 26.5 | 8.5 |
| (f)Ruminococcaceae;(g)Ruminiclostridium 9 | 0 | 0.01 |
| (f)Ruminococcaceae;(g)Ruminiclostridium 5 | 0 | 0 |
| (f)Helicobacteraceae;(g)Helicobacter | 0.01 | 0 |
| (g)anaerobic digester metagenome | 0 | 0 |
| (f)Muribaculaceae;(g)mouse gut metagenome | 0 | 0 |
| (f)Ruminococcaceae;(g)Intestinimonas | 0.01 | 0 |
| (f)Lachnospiraceae;(g)Lachnoclostridium 5 | 0 | 0 |
| (f)Ruminococcaceae;(g)Ruminococcaceae UCG-010 | 0.05 | 0.03 |
| (f)Peptococcaceae;(g)Dehalobacterium | 0 | 0 |
| (f)Barnesiellaceae;(g)Barnesiella | 0.03 | 0.01 |
| (f)Akkermansiaceae;(g)Akkermansia | 0.007 | 0 |
| (f)Ruminococcaceae;(g)Ruminococcaceae UCG-005 | 0 | 0 |
| (f)Streptococcaceae;(g)Streptococcus | 0.2 | 0.09 |
| (f)Ruminococcaceae;(g)Oscillibacter | 0.01 | 0 |
| (f)Ruminococcaceae;(g)Angelakisella | 0 | 0 |
| (f)Christensenellaceae;(g)uncultured | 0 | 0 |
| (f)Ruminococcaceae;(g)Ruminococcaceae UCG-013 | 0 | 0 |
| (f)Muribaculaceae;(g)uncultured bacterium | 0 | 0 |
| (f)Desulfovibrionaceae;(g)Bilophila | 0 | 0 |
| (f)Lachnospiraceae;(g)GCA 900066575 | 0 | 0 |
| (f)Erysipelotrichaceae;(g)Faecalibaculum | 0.01 | 0 |
| (f)Ruminococcaceae;(g)UBA1819 | 0 | 0 |
| (f)Lachnospiraceae;(g)Lachnospiraceae NK4A136 group | 0.17 | 0.003 |
| (f)Lachnospiraceae;(g)Tyzzerella | 0.01 | 0 |
| (f)Atopobiaceae;(g)Coriobacteriaceae UCG-002 | 0 | 0 |
| (f)Muribaculaceae;(g)CAG 873 | 0 | 0 |
| (f)Enterococcaceae;(g)Enterococcus | 0.005 | 0.002 |

**Supplementary Table 1. Mean abundance levels of genera from antibiotic treated mice that were previously significantly altered during HFD.**
