# Supplementary material for: Endothelial IGF‐1 receptor mediates crosstalk with the gut wall to regulate microbiota in obesity
Source: EMBO Rep. 2021 May 2;22(5):e50767. doi: 10.15252/embr.202050767 (PMC8097321; doi:10.15252/embr.202050767)

Figure EV1A

Phospho eNOS (s1177)

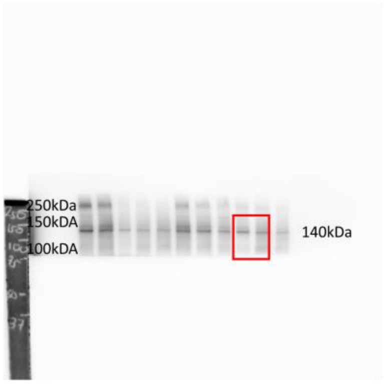

Total eNOS

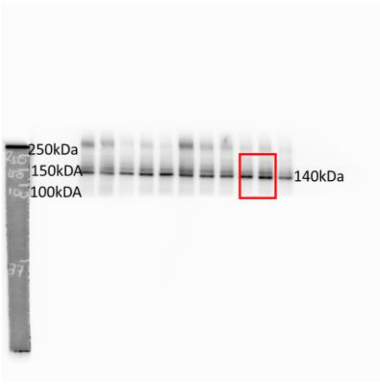

FigureEV1B

p-AKT ser 473

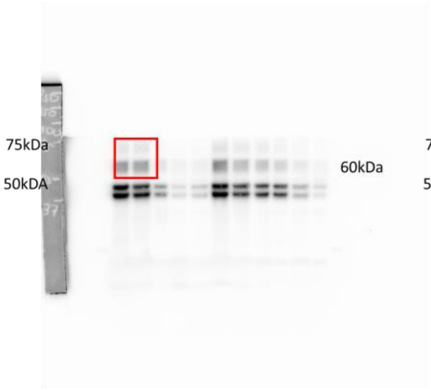

Total AKT

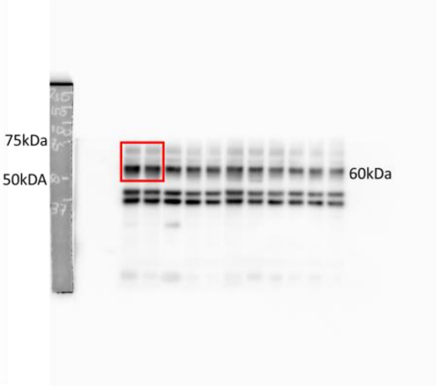

Figure EV1F

AKT

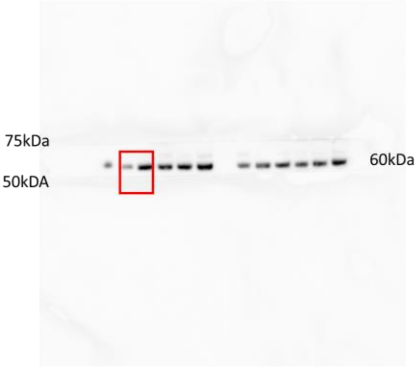

B-actin

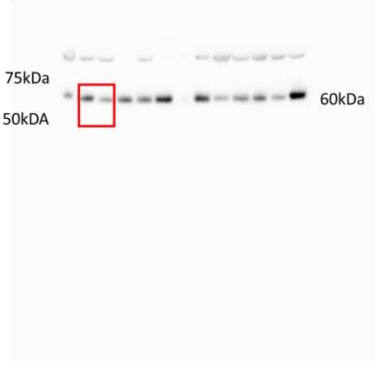

Figure EV1G

p-AKT ser 473

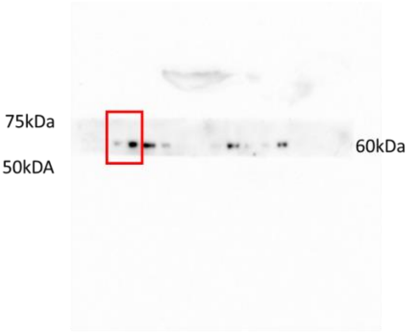

B-actin

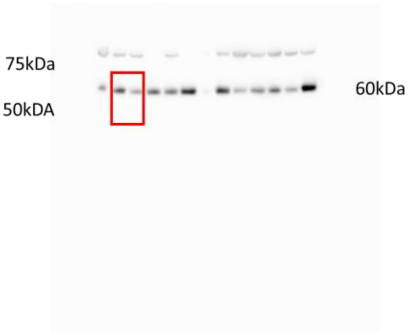

Supplement: Supplementary file 3 — Source Data for Expanded View [file EMBR-22-e50767-s004.zip › embr202050767-sup-0004-SDataFigEV1F.pdf]
