# Supplementary figures and images for: Endothelial IGF‐1 receptor mediates crosstalk with the gut wall to regulate microbiota in obesity
Source: EMBO Rep. 2021 May 2;22(5):e50767. doi: 10.15252/embr.202050767 (PMC8097321; doi:10.15252/embr.202050767)

Figure 1D

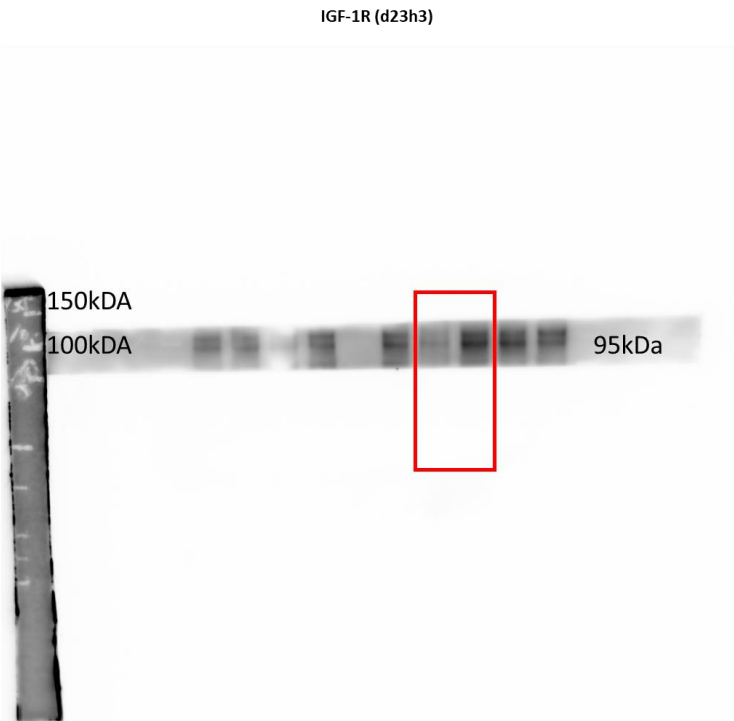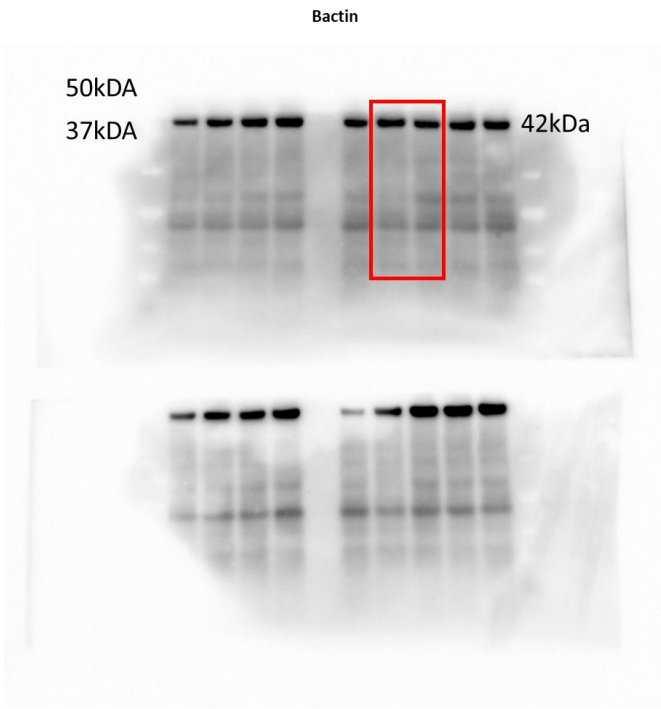

Figure 1E

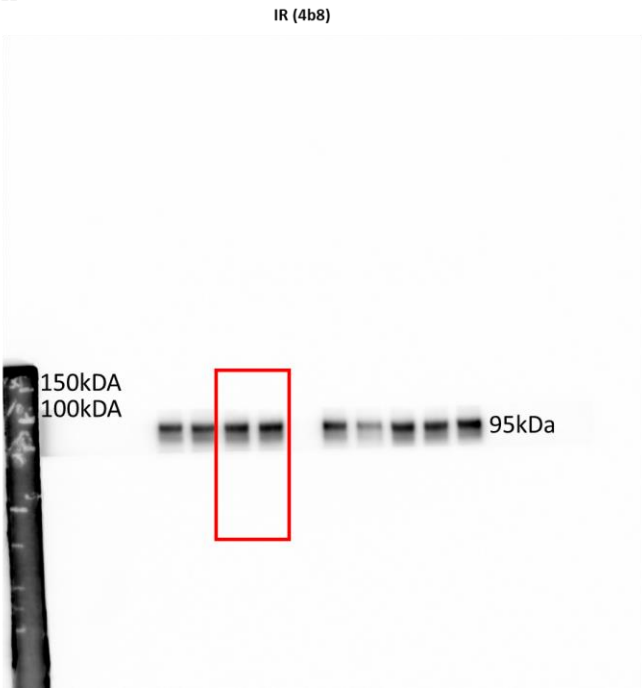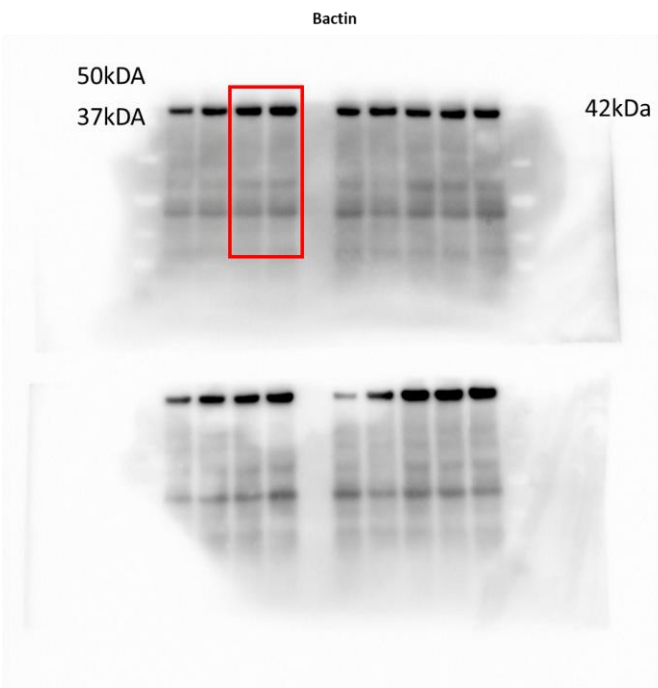

Supplement: Supplementary file 5 — Source Data for Figure 1 [file EMBR-22-e50767-s003.pdf]
